# From Pandemic to COVID-19 Endemic: Mental Health Impact, Psychological and Social Well-Being Among Social Work Students—Implications for the Healthcare System

**DOI:** 10.3390/healthcare13010025

**Published:** 2024-12-26

**Authors:** Alexandru-Cosmin Apostol, Mihaela Rădoi, Gabriela Irimescu

**Affiliations:** Department of Sociology, Social Work and Human Resources, “Alexandru Ioan Cuza” University of Iași, 700506 Iaşi, Romania; mihaela.radoi@uaic.ro (M.R.); gabriela.irimescu@uaic.ro (G.I.)

**Keywords:** COVID-19, social work students, psychological well-being, social well-being, pandemic-related mental health, healthcare

## Abstract

**Background/Objectives:** Our research paper aims to analyse the relationship between the perceived impact on mental health due to the COVID-19 pandemic, social well-being, and psychological well-being among social work students. As we transition from the pandemic phase to an endemic phase of COVID-19, it is necessary to examine these aspects, recognizing the interdependence and complementarity of social and medical care within the healthcare system. **Methods:** The research was conducted nearly three years after the lockdown measures imposed by the spread of COVID-19, during a period when onsite academic activities had resumed, as they were prior to the pandemic. The sample consisted of 235 social work students from both bachelor’s and master’s programmes studying at a large university in Romania. Data was collected between December 2022 and January 2023, during a time when onsite educational activities were once again taking place. **Results:** Our results show that, despite the significant amount of time that had passed since the pandemic began, its negative effects were still strongly felt on a psychosocial level. **Conclusions:** This finding leads us to conclude that there is a long-term COVID-19 impact on mental health. Most study participants reported experiencing high (42.10%) or moderate (23%) levels of being mentally affected, which also affected their psychological and social well-being, key factors in preparing future social-medical care professionals to provide adequate integrated healthcare services.

## 1. Introduction

The COVID-19 pandemic and the subsequent lockdown had a profound impact on human life. Moreover, the pandemic posed significant challenges for higher education institutions dedicated to training future healthcare professionals and caused major disruptions in the teaching and learning processes [1]. The abrupt transition from onsite teaching activities to online learning significantly affected students specialising in social work (SW) or medical fields, primarily because their education heavily relies on practical experiences.

Before the COVID-19 pandemic, studies highlighted that social work education is known for its high emotional and psychological demands. Social work students face not only complex academic tasks but also practical situations involving direct interactions with clients in challenging situations [2]. Other studies report that the stressful experiences during these students’ internships are often associated with emotional exhaustion and lower personal satisfaction [3].

Following the outbreak of the COVID-19 pandemic, the challenges faced by social work students intensified, particularly concerning their mental health, social well-being, and psychological well-being. According to the European Commission [4], four major dimensions related to sustainable development goals in education focus on psychological, social, cognitive, and physical well-being. Therefore, this article aims to explore two of these dimensions—psychological well-being and social well-being—in relation to students’ self-reported mental health impact caused by the COVID-19 pandemic.

Mental health is a dynamic state of internal equilibrium that enables individuals to use their abilities in harmony with the universal values of society. Basic cognitive and social skills; ability to recognise, express and modulate one’s own emotions, as well as empathise with others; flexibility and ability to cope with adverse life events and function in social roles; and harmonious relationship between body and mind represent important components of mental health which contribute, to varying degrees, to the state of internal equilibrium [5].

Social well-being refers to the quality of interpersonal relationships and an individual’s ability to integrate and function effectively in society [6]. It encompasses dimensions such as social actualisation, social acceptance, social integration, social contribution, and social coherence [6]. In addition, psychological well-being involves integrating two essential aspects of life (i.e., the combination of feeling good and functioning efficiently) [7,8]. The concept of effective functioning (in a psychological sense) includes developing one’s potential, having control over one’s life, having a purpose (e.g., working to achieve valuable goals), and experiencing positive relationships. Psychological well-being, characterised by feelings, cognitions, and strategies associated with positive functioning, has been linked to better physical health and greater longevity. Significantly, various interventions can enhance psychological well-being by providing a strategy to improve population health [7].

Our research paper aims to analyse the relationship between the perceived impact on mental health due to the COVID-19 pandemic, social well-being, and psychological well-being among social work students. Another objective pursued through this research was to identify the proportion of students who self-reported being severely or moderately mentally affected by the COVID-19 pandemic.

## 2. Background

### 2.1. The Relationship Between Self-Assessed Mental Impact of COVID-19, Social Well-Being, and Psychological Well-Being

Since 2020, students have reported increasingly high levels of uncertainty regarding their academic future, significant stress levels, and struggles to cope with disruptions caused by COVID-19 [9]. Additionally, students experienced social distancing, remote learning, and changes in daily life, all leading to higher levels of stress and anxiety, with significant effects on mental health [10]. Consequently, the substantial discomfort felt by students during lockdown due to the challenges of academic adaptation and feelings of social isolation contributed most to increased emotional difficulties and decreased well-being [11,12].

Studies have proven that low well-being levels can contribute to the onset or worsening of mental disorders [13], while chronic stress can entail emotional exhaustion and a negative self-perception of life [14]. Furthermore, research shows that individuals experiencing mental disorders are more likely to report lower levels of psychological well-being [15].

Focusing on students, particularly those potentially vulnerable from a psychosocial perspective, Van Eekert et al. [16] explored how the pandemic affected their mental health. The study’s findings indicate that the lack of social interactions and support networks severely impacted students’ mental well-being. The restrictions enforced, social isolation, and difficulties with academic adaptation intensified the mental health impact of the COVID-19 pandemic, thus negatively affecting personal satisfaction and emotional balance. Social work students were particularly affected, given their demanding academic requirements and the challenges of completing their internships in highly difficult contexts [17].

Similar studies highlight the significant impact of the COVID-19 pandemic on students’ psychological well-being [18,19,20]. Pre-pandemic positive psychology research suggests that high levels of subjective psychological well-being are associated with numerous benefits (i.e., increased longevity, better health, and greater life efficiency) [21]. They are particularly relevant in educational settings, where such outcomes can influence teaching and learning experiences alike. Students experiencing positive emotions are more prone to have better focus, higher levels of creativity, and enhanced problem-solving skills, all of which contribute to improved academic performance. Psychological well-being can act as a protective factor against mental health issues. Emotional resilience, a critical component of well-being, is associated with reduced stress impact and an increased capacity for recovery when facing adversities [22].

Subjective well-being, closely related to psychological well-being, refers to an individual’s self-assessment, including feelings of happiness, satisfaction, and emotional balance [23]. The relationship between subjective psychological well-being and social well-being is bidirectional and complex. Studies show that individuals with strong social support are prone to higher psychological well-being levels [24]. Participation in social activities can provide opportunities for personal development and self-esteem improvement, contributing to a positive mental state [25]. On the other hand, positive interpersonal relationships can foster a sense of belonging and security, thus mitigating the risk of social isolation and mental disorders [26].

At the same time, individuals with high social well-being are more likely to develop and maintain healthy social relationships and actively participate in their communities [6]. They are more open to social interactions, better able to manage interpersonal conflicts, more resilient to stress, and more capable of handling negative emotions [27]. A positive psychological state can also enhance communication and empathy skills, thus facilitating healthy social relationships [28].

Undoubtedly, how universities operate in the post-pandemic scenario cannot be considered entirely normal [29]. The events and experiences during part of the 2019–2020 academic year and throughout 2020–2022 marked a significant turning point in the awareness and consideration of emotional and mental health for all members of the educational community and their impact on learning outcomes.

Given all the elements above, studying and monitoring the well-being of social work students and the ways in which their well-being was affected during the COVID-19 pandemic is particularly relevant. It is well-known that social workers are at increased risk of fatigue due to the nature of their work and the challenges posed by the needs of various types of clients [30]. They are also prone to burnout from occupational stress [31] and secondary trauma [32,33]. Therefore, the ability to effectively manage emotional responses, often in complex healthcare contexts, is essential for the role of a future professional in the social work field.

### 2.2. Social Work—An Integral Part of the Healthcare System

The context of the COVID-19 pandemic, with its effects as defined by the WHO [34] as large-scale foci of infectious diseases significantly increasing morbidity and mortality over a wide geographical area, the secondary effects of which include substantial economic, social, and political disruptions, underscores the importance of social workers, their role, and their involvement in disaster response, humanitarian emergencies, and pandemics. Due to their professional responsibility to enhance individual well-being and holistically support vulnerable populations, social workers are regarded as a valuable group of healthcare professionals endowed with adequate knowledge and skills for intervention in post-disaster recovery contexts [35,36]. From a historical perspective, the profession of social work is deeply rooted in disaster assistance and post-disaster intervention [37,38]. Payne [39], while gaining extensive and globally recognised expertise in the field of social work, emphasises that his vision of this field of healthcare professionals played a significant role in broadening his personal view. Against such a background, Payne mentions that one of the three important management settings in which social work has developed includes: “hospitals and municipal services provided through agencies whose main purpose, for example, education, healthcare, and housing, was not social provision” [39] (p. 106).

Undoubtedly, social care is integrated and delivered through social work professionals within the healthcare delivery system, as highlighted in studies and reports focused on analysing social risk factors and the contexts in which they emerge [40,41,42]. The definition of health as a dynamic state of well-being, characterised by a physical and mental condition meeting the life requirements of the individual in relation to age, culture, and personal responsibility [43], as an essential element of human well-being [44], accounting for a value in itself and a significant component of human capital based on the fundamental rights of individuals [45] underscores the role of social service specialists in maintaining, improving, or enhancing health. Social risk factors must be considered when discussing improving primary prevention and treatments for acute and chronic conditions [39].

Relying on this premise, the connection between social work practice and the healthcare system can be illustrated from the perspective of the thirteen standards developed by the National Association of Social Workers [46]. The evolution of the view of social work as an integral part of the healthcare system is becoming increasingly prominent at an international level, as professional social workers play an essential role in the medical field, working in relevant institutions and organisations (hospitals, oncology departments, emergency departments, neonatal units, palliative care centres, medico-social centres, home care centres, rehabilitation centres, mental health centres, etc.).

The classification provided by the World Health Organisation for healthcare professionals [47], relying on the International Standard Classification of Occupations, includes social workers within the occupation group “Health management and support personnel”. According to the classification under the code ISCO 2635, “*Social work and counselling professionals provide counselling, therapy and mediation services to individuals, families, groups and communities in response to social and personal difficulties. They assist clients to develop skills and access resources and support services needed to respond to issues arising from health problems, life transitions, addictions, and other personal, family and social problems. They liaise with other social service agencies, educational institutions and health care providers to advocate for client and community needs*” [47] (p. 12). We add Payne’s perspective, who argues the following “*social work as seeking the best possible well-being for individuals, groups and communities in society, by promoting and facilitating growth and self-fulfilment*” [39] (p. 12).

In Romania, according to the report drafted as part of the project “Strengthening the Labour Force in Social Work Services in Romania”, conducted between 2018 and 2019 with the support of the local UNICEF branch and the contribution of the National College of Social Workers in Romania, the connections between the social worker profession and the healthcare system are apparent. This profession is present in one form or another within the medical sector [48]. In this context, the European Skills/Competences, Qualifications, and Occupations (ESCO) framework is mentioned, highlighting that, based on training obtained through a university programme, various specialities related to the healthcare system can be found at the European level. They include social workers specialising in mental health, medico-social social workers, psychiatric social workers, or social workers in palliative care [48] (p. 34). Social work university studies provide comprehensive skills and competencies, including working in a multicultural environment in healthcare, implementing evidence-based decision-making in medical assistance, engaging with the social network of healthcare users, collaborating within multidisciplinary medical teams, organising medical services for patients at home, advocating for the needs of healthcare users, and complying with healthcare legislation [48] (pp. 38–44). The current legislation framework governing the provision of social services in Romania emphasises an interdisciplinary approach, acknowledging the involvement of multiple personnel categories in social protection activities.

According to data provided by the Centre for Research on the Epidemiology of Disasters [49], an increasing trend in the frequency of natural disasters (biological, climatological, meteorological, hydrological) has been recorded since the 1960s. However, nothing has highlighted the need for society to focus on disaster risk mitigation as clearly as the COVID-19 pandemic. This crisis revealed numerous deficiencies, including gaps in global preparedness for pandemics in areas such as detection, basic healthcare availability, contact tracing, quarantine, and isolation procedures, as well as training specialists in the medical sector and social support services [50]. Social workers, alongside medical personnel, are on the front lines during pandemics [51], and the response of social services and the healthcare system to the COVID-19 pandemic has entailed an overload of professionals within these fields.

The pandemic radically altered many aspects of life and livelihoods in the first half of 2020. The conditions worsened for beneficiaries of social work services, who were already in vulnerable situations, that is, individuals on the margins of society suffering from poor health, poverty, racism, and other forms of oppression and inequality. Social isolation increased, jobs disappeared, and access to several socio-medical services was restricted. As Amadasun notes [52], the pandemic negatively impacted the fundamental values of social work (promoting human rights, the right to self-determination and participation, promoting social justice, respecting the dignity and worth of individuals, the relevance of human relationships, etc.).

Social workers struggled to intervene, adapting and innovating to meet new needs and reprioritising the most urgent and significant aspects of their roles [53].

In conclusion to this part, it is worth noting that regardless of the historical period or geographic region, health crises significantly impact the educational process/system, particularly those segments preparing future specialists in the social and medical care field. This makes investigating the well-being levels reported by social work students all the more necessary.

## 3. Materials and Methods

### 3.1. Participant Recruitment

A standardised, self-administered questionnaire was used for data collection via the Google Forms platform, with the research being cross-sectional. The period for collecting answers spanned from December 2022 to January 2023, during which 235 social work students responded to our invitation to participate in the study. This period coincided with the resumption of onsite university courses after 2.5 years of primarily online academic activities due to restrictions imposed by the evolution of the COVID-19 pandemic.

### 3.2. Data Collection and Instruments

When designing the questionnaire, we focused on three dimensions related to the situation of social work students, measuring the following parameters: the level of self-assessed impact on mental health due to the COVID-19 pandemic (1 item), the level of social well-being (15 items), and the level of psychological well-being (8 items).

To measure the level of perceived impact on mental health among students regarding the COVID-19 pandemic, a scaled question was formulated as follows: “*Generally, on a scale from 1 to 10, how much do you think you were mentally affected by the situation of the past year caused by the evolution of the COVID-19 pandemic?*”. The scores for this item ranged from a minimum of 1 (not at all), indicating respondents who reported no psychological impact due to the pandemic, to a maximum of 10 (extremely), indicating respondents who reported being extremely affected psychologically by the effects of COVID-19.

Social well-being was measured using an instrument proposed by Keyes [6]. The Social well-being scale is widely used in specialised studies, having been previously validated in various contexts, with numerous researchers already demonstrating its psychometric properties and reliability in application [54,55,56]. For the purposes of our study, we used the short version of the Social Well-Being Scale, which includes a total of 15 items measuring indicators related to social integration (i.e., I do not feel I belong to anything I’d call a community), social acceptance (i.e., People who do a favour expect nothing in return), social contribution (i.e., I have something valuable to give to the world), social actualisation (i.e., The world is becoming a better place for everyone), and social coherence (i.e., The world is too complex for me). Each item was measured using a 7-point scale ranging from 1 (strongly disagree) to 7 (strongly agree). Therefore, the total scores calculated for assessing overall social well-being could range from a minimum of 15 to a maximum of 105. In the analysis, the scores for negative items were reversed. Ultimately, high scores indicated a higher level of social well-being, while low scores reflected a lower level of social well-being.

To assess the psychological well-being, we used the Flourishing Scale [8]. Since its introduction, the Flourishing scale has become an extensively used scale internationally, and its validity has been demonstrated numerous times in comprehensive studies conducted previously [57,58,59,60]. This scale includes eight items, with each statement being rated with scores ranging from a minimum of 1 (strongly disagree) to 7 (strongly agree). Consequently, the total scores could range from a minimum of 8, indicating a low level of psychological well-being, to a maximum of 56, reflecting a high level of well-being among the students who participated in the study.

The items included in the Social Well-Being Scale and the Flourishing Scale were translated from English into Romanian by a certified translator.

### 3.3. Statistical Analysis

We used IBM^®^ SPSS^®^ Statistics 26.0.0 in the data processing and analysis process. We calculated the frequencies, percentages, means, medians, and standard deviations depending on the nature of the variables analysed. We applied the Independent Samples T-Test to highlight differences between subgroups within the population studied. The population subgroups studied were formed based on their socio-demographic characteristics: gender (male and female), level of study (undergraduate and master’s), and area of residence (urban and rural). We calculated the correlations between the scores assigned to the applied scales using the Pearson Correlation Coefficient. We took into account a significance threshold of *p* < 0.05 to indicate statistically significant differences.

### 3.4. Ethical Statement

Participation in the research was voluntary, with students being informed through an informed consent form about the primary details of the study. From the very beginning of the questionnaire, students could choose whether to participate. All answers were confidential and anonymous, with no personal data requested that could enable identifying respondents or performing individual analyses. Lastly, the research study was conducted in accordance with the principles outlined by the Declaration of Helsinki [61].

## 4. Results

The following lines feature the primary research findings, considering the correlations between the aforementioned measurements and the analyses performed to identify statistically significant differences between the subgroups of the population analysed in this study.

### 4.1. Sociodemographic Characteristics of the Group

The sample comprised 235 social work students within “Alexandru Ioan Cuza” University in Iași, including both undergraduate students (*n* = 189; 80.40%) and Master’s students (*n* = 46; 19.60%). All students included in the sample had experienced at least one year of studies during the COVID-19 pandemic restrictions, thus excluding first-year undergraduate students (Table 1).

A characteristic of the gender distribution, consistent with the structure of the population studied, was the predominance of female students (*n* = 218; 92.80%). Additionally, just over two-thirds of the respondents reported residing in urban areas (*n* = 158; 67.20%), while the rest indicated living in rural areas (*n* = 77; 32.80%). The mean age of respondents was 24.21 (*SD* = 8.13), which was higher among Master’s students (*M* = 27.20; *SD* = 8.53) compared to undergraduate students (*M* = 23.48; *SD* = 7.88).

### 4.2. The Correlation Between Perceived Impact of the COVID-19 Pandemic on Mental Health (MI), Social Well-Being (SWB), and Psychological Well-Being (PSWB)

Through the reliability analyses carried out, the social well-being and the flourishing scale benefit from high scores of Cronbach’s alpha coefficient (α SWB = 0.845; α PSWB = 0.921) (Table 2).

At the same time, we highlighted the association between the degree of perceived impact of the COVID-19 pandemic on mental health (MI), social well-being (SWB), and psychological well-being (PSWB) using Pearson’s correlation. Following data analysis, we have found statistically significant correlations that can be interpreted as follows:

- The students who declared having been mentally affected by the effects of the COVID-19 pandemic reported lower levels of social well-being (*r* = −0.209; *p* < 0.01) and psychological well-being (*r* = −0.181; *p* < 0.01);
- The students who manifest a high level of social well-being are characterised by high levels of psychological well-being (*r* = 0.699; *p* < 0.01).

### 4.3. Self-Reported Perceived Impact of the COVID-19 Pandemic on Mental Health Among Social Work Students

Across the entire population included in the research sample, the perceived level impact of the COVID-19 pandemic on mental health among the students investigated was moderate (*M* = 5.53; *Me* = 6.00; *SD* = 2.86). Based on the collected data, we found that the proportion of social work students who self-reported a high level of perceived impact of the COVID-19 pandemic on mental health—scores of 7 or above—was 42.10%, considerably higher than those who reported low scores in the 1–4 range (34.90%). Those who selected median scores of 5 and 6 accounted for 23% of the total responses analysed (Table 3).

### 4.4. Descriptive Analysis Regarding the Perceived Impact of the COVID-19 Pandemic on Mental Health Among Social Work Students (Mean, Median, and Standard Deviation)

Analysing the mean scores calculated based on various sociodemographic variables, we identified that, from a descriptive standpoint, female students (*M* = 5.66; *SD* = 2.77), students from rural areas (*M* = 5.65; *SD* = 2.93), and those pursuing undergraduate studies in social work (*M* = 5.57; *SD* = 2.92) self-reported slightly higher levels of perceived impact of the COVID-19 pandemic on mental health in the year preceding the research, compared to male students (*M* = 3.88; *SD* = 3.58), urban residents (*M* = 5.47; *SD* = 2.84), and the subgroup of respondents in Master social work programmes (*M* = 5.35; *SD* = 2.62).

Using the Independent Samples T-Test, we found that the perceived impact of the COVID-19 pandemic on mental health was significantly higher among female students (*t*(233) = −2.48; *p* = 0.014). For the other cases analysed, no statistically significant differences were observed based on the level of study or place of residence (Table 4).

### 4.5. Social Well-Being of Social Work Students (Mean, Median, and Standard Deviation)

Concerning the level of social well-being measured, the research study revealed higher scores recorded among male students (*M* = 74.17; *SD* = 14.86), Master’s students (*M* = 75.08; *SD* = 12.92), and those living in rural areas (*M* = 72.62; *SD* = 13.23). However, considering the results of the Independent Samples T-Test, the social well-being level was significantly higher only for students enrolled in Master programmes (*M* = 75.08; *SD* = 12.92) compared to those pursuing Bachelor studies in social work (*M* = 68.84; *SD* = 14.01), *t*(233) = −2.75; *p* = 0.006. No statistically significant differences were calculated in the other cases analysed (Table 5).

### 4.6. Psychological Well-Being of Social Work Students (Mean, Median, and Standard Deviation)

Just like for social well-being measurement, in what concerns psychological well-being, measured using the Flourishing scale, slightly higher scores were found among male students (*M* = 45.23; *SD* = 9.51), Master’s students (*M* = 46.28; *SD* = 7.75), and the population residing in rural areas (*M* = 45.10; *SD* = 8.48). Upon applying the Independent Samples T-Test, the only statistically significant difference regarding psychological well-being can be highlighted for the population subgroups divided by study cycle. Therefore, the Master’s students had a higher psychological well-being level (*M* = 46.28; *SD* = 7.75) compared to Bachelor’s students (*M* = 42.99; *SD* = 9.29), *t*(233) = −2.22; *p* = 0.028. No statistically significant differences were found considering the sociodemographic variables regarding the gender and residence area of the respondents (Table 6).

## 5. Discussion

The year 2020 was, without a doubt, a turning point in recent global history. Amid the onset of the COVID-19 pandemic, both educational and healthcare systems were forced to respond rapidly to the new challenges emerging in society. Students preparing for careers in the integrated field of healthcare were exposed to a heightened risk of mental health deterioration and poor emotional well-being due to the pandemic, underscoring the urgent need for intervention and the implementation of adequate strategies to prevent well-being from lowering, a need highlighted in previous studies as well [9].

As noted throughout this paper, efforts to emphasise the close connection between the social care system and the healthcare system have a relatively rich history and, more significantly, precede the onset of the COVID-19 pandemic [62,63,64]. The global trend is undoubtedly moving toward recognising that social work is a relevant and complementary component of the healthcare system. This is apparent in the associated professions intrinsically linked to the medical field, professions highlighted earlier in this paper and toward which some young graduates of social work programmes aspire to transition.

For this reason, this paper sought to underline how social work students were mentally affected, correlating it with the impact on two essential components of individual well-being: social and subjective psychological well-being. We began with the premise that social work students, through the skills and competencies acquired during their university training, can contribute to improving individual well-being, thereby facilitating the mission of healthcare professionals. Consequently, it would be interesting to find the answer to the following question in future studies: To what extent are social work students prepared to deliver well-being in medical settings, given that they experience the adverse effects of the COVID-19 pandemic on their mental health, with implications for their well-being?

This question arose against the backdrop of a gap in the literature, which has not yet addressed this topic, particularly in the local context. It is worth noting that interest in analysing the mental health of social work students predates the COVID-19 pandemic, given that these students train to face a career in a highly demanding psychosocial field [65,66].

In addition, to mitigate the pressure exerted on healthcare professionals, specialists also need to focus on the social determinants of health (i.e., the social workers) [67]. The link between the two categories of specialists is the “well-being” of the beneficiaries of integrated healthcare services, which encompass both medical and social care.

For medical specialists collaborating with social work professionals, it is critical to understand the latter’s profile, especially since the degree of well-being impairment caused by the COVID-19 pandemic is significant. We believe that compensating for practical and fieldwork activities, such as direct work with beneficiaries, a mandatory requirement for future integrated healthcare system specialists, can be achieved through multiple channels. We refer to permanent professional development programmes, lifelong learning for students and recent graduates targeting skills and competencies they could not acquire or develop during the pandemic, and technology-based clinical simulations to enhance clinical skill training [68]. Additionally, creating professional development opportunities to improve direct practice skills post-pandemic is essential.

The analysis results indicate that the long-term COVID-19 phenomenon is manifesting in the mental health of social work students preparing for professionalisation in the field, though data collection coincided with a study period that resembled the pre-COVID-19 era in its characteristics. Notably, two and a half years after declaring the COVID-19 pandemic, the proportion of students reporting a high degree of being mentally affected exceeds that of those who reported lower levels. This finding is consistent with other studies conducted post-2022, indicating that COVID-19-related mental health impact persisted despite considerable efforts to resume “normal” life as it was known and experienced before the pandemic [69,70]. Furthermore, Uclaray’s study [71] underlines that integrating topics related to stress prevention and self-care promotion into the social work curriculum and supervision can help students who train in social care to manage better professional stress, academic responsibilities, and personal challenges. This approach could lead to the development of future social workers who understand how to maintain healthy well-being, making them more effective, competent, and ethical practitioners prepared to collaborate with healthcare professionals.

In such a context, our findings show that students who self-reported a significant mental impact from the pandemic tend to report lower levels of social well-being and psychological well-being. These two components are strongly intertwined. Therefore, it is apparent that there is a long-term mental health impact within the population investigated, primarily attributed to the psychosocial factors associated with COVID-19. This issue has also been predicted and analysed in the literature [72].

The cross-sectional design of the study represents one of the research’s limitations. This prevents us from comparing data over a longer period of time within the same population of students. Additionally, although the population of social work students is predominantly female, a fact reflected in the characteristics of the studied population, we consider this another limitation, as this gender imbalance may make the data difficult to generalise. Furthermore, the research was conducted on a population whose studies had a specific context during the COVID-19 pandemic, different from other international contexts, and therefore may represent a limitation of our study, with implications for the generalisation of the results.

## 6. Conclusions

In this paper, we have focused on a specific category of students preparing for professional careers in social work, a field strongly interconnected with healthcare. Based on our experience, we can state that social work students frequently face intense academic demands and exposure to emotionally challenging contexts, which can lead to diminished mental health and psychological and social well-being.

Thus, we highlighted that nearly three years after enforcing lockdown measures due to the COVID-19 pandemic, the negative consequences were still sensed in terms of mental health, with repercussions on psychological and social well-being. With four out of ten students reporting high levels of being mentally affected and two out of ten reporting moderate levels, it is clear that COVID-19 has become endemic. Social distancing measures and restrictions on daily interactions worsened social isolation, and the lack of opportunities for direct contact with healthcare recipients in practice centres also took a toll on the mental state of social work students.

Moreover, it became apparent that the three elements of individual well-being presented throughout the paper are closely interconnected and significantly influence one another. As a practical implication for the future, we consider that promoting an active and healthy social life can contribute to improved mental health. At the same time, preserving a positive psychological state can facilitate social relationships and community participation. In the context of the academic and professional lives of social work students during the pandemic, recognising and addressing these interdependencies is essential for promoting well-being.

The COVID-19 pandemic has only highlighted the psychosocial vulnerabilities of social work students and emphasised the relevance of appropriate educational interventions. Developing a curriculum that includes mental health support and promotes well-being is essential to shape a resilient generation of social care professionals. Studying well-being is a dynamic mission, mainly when focusing on a specific population group like students, as the effects of low well-being levels are not limited to the short term. Thus, without investing in improvement, low well-being levels can impact academic achievement, transition into adulthood, and healthcare labour force integration.

In our view, an increasing number of studies will continue to underline the social and economic impact of the COVID-19 pandemic on students, social workers, and healthcare specialists. Considering these aspects, there is an urgent need to support students in developing skills through joint programmes between universities and medico-social institutions, thus ensuring high-quality jobs or internships and flexible practicum options. The ability to adapt to change should be an essential characteristic of the profession, as well as of students and professionals in the socio-medical care field.

Therefore, it is necessary to learn from the pandemic experience, seek new strategies, and take into account aspects to improve in the near future.

## Figures and Tables

**Table 1:** Socio-demographic data of the respondents.

Sociodemographic Variable	*n*	% or Mean (Std. Dev.)
Gender		
Male	17	7.2%
Female	218	92.8%
Academic cycle		
Bachelor’s degree Social Work (BSW)	189	80.4%
Master studies Social Work (MSW)	46	19.6%
Year of study BSW		
Second year	98	51.9%
Third year	91	48.1%
Year of study MSW		
First year	35	76.1%
Second year	11	23.9%
Area of residence		
Urban	158	67.2%
Rural	77	32.8%
Age of respondents	233 *	24.21 years old (8.13)
Age of respondents BSW	187	23.48 (7.88)
Age of respondents MSW	46	27.20 (8.53)

* Two respondents did not state their age.

**Table 2:** Correlation between the perceived impact of the COVID-19 pandemic on mental health (MI), social well-being (SWB), and psychological well-being (SWB) and reliability analysis.

Scale (n = 235)	[1]	[2]	[3]	Min.	Max.	*M*	Std. Dev.	α
[1] MI (1 item)		−0.209 **	−0.181 **	1	10	5.53	2.86	N/A
[2] SWB (15 items)			0.699 **	15	105	70.06	13.99	0.845
[3] PSWB (8 items)				8	56	43.63	9.09	0.921

** *p* < 0.01; *M* = Mean; Std. dev. = Standard deviation; α = Cronbach’s alpha coefficient.

**Table 3:** Self-reported perceived impact of the COVID-19 pandemic on mental health among social work students.

Generally, on a Scale from 1 to 10, How Much Do You Think You Were Mentally Affected by the Situation of the Past Year Caused by the Evolution of the COVID-19 Pandemic?	% (*n* = 235)	Mean [*M*]	Median [*Me*]	Standard Deviation [*SD*]
1 [Not at all]	14.50%	5.53	6.00	2.86
2	7.20%			
3	4.30%			
4	8.90%			
5	13.20%			
6	9.80%			
7	11.50%			
8	14.00%			
9	8.10%			
10 [Extremely]	8.50%			

**Table 4:** Differences in perceived impact of the COVID-19 pandemic on mental health in relation to gender, academic cycle, and area of residence.

Sociodemographic Variable	*M*	*Me*	*SD*	*t*
Gender				
Male	3.88	2.00	3.58	*t*(233) = −2.48; *p =* 0.014 *
Female	5.66	6.00	2.77	
Academic cycle				
Bachelor’s degree Social Work (BSW)	5.57	6.00	2.92	*t*(233) = 0.51; *p =* 0.636
Master studies Social Work (MSW)	5.35	6.00	2.62	
Area of residence				
Urban	5.47	6.00	2.84	*t*(233) = −0.45; *p =* 0.651
Rural	5.65	6.00	2.93	

* *p* < 0.05; *M* = Mean; *Me* = Median; *SD* = Standard deviation.

**Table 5:** Differences in social well-being in relation to gender, academic cycle, and area of residence.

Sociodemographic Variable	*M*	*Me*	*SD*	*t*
Gender				
Male	74.17	81.00	14.86	*t*(233) = 1.26; *p =* 0.210
Female	69.74	70.00	13.91	
Academic cycle				
Bachelor’s degree Social Work (BSW)	68.84	68.00	14.01	*t*(233) = −2.75; *p =* 0.006 **
Master studies Social Work (MSW)	75.08	74.50	12.92	
Area of residence				
Urban	68.82	68.00	14.22	*t*(233) = −1.97; *p =* 0.051
Rural	72.62	72.00	13.23	

** *p* < 0.01; *M* = Mean; *Me* = Median; *SD* = Standard deviation.

**Table 6:** Differences in psychological well-being in relation to gender, academic cycle, and area of residence.

Sociodemographic Variable	*M*	*Me*	*SD*	*t*
Gender				
Male	45.23	47.00	9.51	*t*(233) = 0.75; *p =* 0.453
Female	43.51	45.00	9.07	
Academic cycle				
Bachelor’s degree Social Work (BSW)	42.99	45.00	9.29	*t*(233) = −2.22; *p =* 0.028 *
Master studies Social Work (MSW)	46.28	47.00	7.75	
Area of residence				
Urban	42.92	45.00	9.31	*t*(233) = −1.73; *p =* 0.085
Rural	45.10	46.00	8.48	

* *p* < 0.05; *M* = Mean; *Me* = Median; *SD* = Standard deviation.

## Data Availability

Data are contained within the article.
